# HIV self‐testing intervention experiences and kit usability: results from a qualitative study among men who have sex with men in the SELPHI (Self‐Testing Public Health Intervention) randomized controlled trial in England and Wales

**DOI:** 10.1111/hiv.12818

**Published:** 2019-12-10

**Authors:** TC Witzel, A Bourne, FM Burns, AJ Rodger, L McCabe, MM Gabriel, M Gafos, D Ward, Y Collaco‐Moraes, DT Dunn, A Speakman, C Bonell, R Pebody, FC Lampe, J Harbottle, AN Phillips, S McCormack, P Weatherburn

**Affiliations:** ^1^ Department of Public Health, Environments and Society Faculty of Public Health and Policy London School of Hygiene and Tropical Medicine London UK; ^2^ Australian Research Centre in Sex, Health and Society La Trobe University Melbourne VIC Australia; ^3^ Institute for Global Health University College London London UK; ^4^ Medical Research Council Clinical Trials Unit University College London London UK; ^5^ Department of Global Health and Development Faculty of Public Health and Policy London School of Hygiene and Tropical Medicine London UK; ^6^ NAM Aidsmap London UK; ^7^ SH:24 London UK

**Keywords:** HIV prevention, HIV testing, implementation science, men who have sex with men, process evaluation

## Abstract

**Objectives:**

SELPHI (HIV Self‐Testing Public Health Intervention) is the largest randomized controlled trial (RCT) of HIV self‐testing (HIVST) in a high‐income setting to date, and has recruited 10 000 men who have sex with men (cis‐ and transgender) and transgender women who have sex with men. This qualitative substudy aimed to explore how those utilizing self‐tests experience HIVST and the implications for further intervention development and scale‐up. This is the first qualitative study in Europe investigating experiences of HIVST among intervention users, and the first globally examining the experience of using blood‐based HIVST.

**Methods:**

Thirty‐seven cisgender MSM SELPHI participants from across England and Wales were purposively recruited to the substudy, in which semi‐structured interviews were used to explore testing history, HIVST experiences and intervention preferences. Interviews were audio‐recorded, transcribed and analysed through a framework analysis.

**Results:**

Men accessed the intervention because HIVST reduced barriers related to convenience, stigma and privacy concerns. Emotional responses had direct links to acceptability. Supportive intervention components increased engagement with testing and addressed supportive concerns. HIVST facilitated more frequent testing, with the potential to reduce sexually transmitted infection (STI) screening frequency. Substudy participants with an HIV‐positive result (*n* = 2) linked to care promptly and reported very high acceptability. Minor adverse outcomes (*n* = 2; relationship discord and fainting) did not reduce acceptability. Ease of use difficulties were with the lancet and the test processing stage.

**Conclusions:**

Intervention components shaped acceptability, particularly in relation to overcoming a perceived lack of support. The intervention was broadly acceptable and usable; participants expressed an unexpected degree of enthusiasm for HIVST, including those with HIV‐positive results and individuals with minor adverse outcomes.

## Introduction

Reducing the prevalence of undiagnosed HIV infection is a key public health goal 1, 2 enshrined in the Joint United Nations Programme on HIV/AIDS (UNAIDS) 90‐90‐90 targets; 90% of people with HIV infection diagnosed, 90% of people diagnosed on treatment and 90% of people on treatment achieving virological suppression 1. The UK has been successful in this regard, with London the first city globally to achieve 95‐95‐95 3. While the HIV incidence in England is falling in gay men, expanding testing remains a priority, with prompt diagnoses and linkage into clinical services for those testing positive 4, 5. Despite the British HIV Association recommending annual testing for men who have sex with men (MSM) (or more frequently if at ongoing risk) 6, up to 25% of gay men and bisexual men have never tested and approximately half have not tested in line with these guidelines 7, 8.

HIV self‐testing (HIVST) is an approach whereby an individual uses a rapid diagnostic test and interprets their own result. HIVST has the potential to increase testing by providing convenience, privacy and accessibility 9, 10. The World Health Organization (WHO) recommends HIVST as a testing option 11.

HIV self‐testing was legalized in the UK in 2014, with the first test coming to market in 2015 10, 12, 13. Widespread free public provision of HIVST has yet to occur in the UK. Pilot and demonstration projects have delivered a limited number of free tests, mainly to MSM and black African people 14, 15, 16.

Acceptability studies have focused on *potential* users of HIVST, with limited numbers of actual self‐testers included in these formative studies 10, 13, 17. Evidence of actual user experience from the UK and from Europe more broadly is limited 18.

SELPHI (HIV Self‐Testing Public Health Intervention) is the largest randomized controlled trial (RCT) of HIVST in a high‐income setting to date. SELPHI has recruited 10 000 MSM (both cisgender and transgender) and transgender women who have anal sex with men. SELPHI has two randomizations and two versions of the primary intervention. In randomization A, the 10 000 participants were allocated at a ratio of 60:40 to a single HIVST (intervention A) at baseline versus standard of care (SoC) (signposting to free testing services). In randomization B (which occurred 3 months post enrolment), eligible participants who had been allocated to HIVST in randomization A, remained HIV‐negative and reported condomless anal intercourse (CAI) in the preceding 3 months were randomized 50:50 to the offer of 3‐monthly repeat HIVST with test reminders (intervention B) versus SoC. SELPHI has recruited from geolocation hook‐up applications (apps) and from social media. All RCT data collection was online, and blood‐based test kits (BioSure (UK) Ltd, Nazeing, UK) were delivered by post directly from the test manufacturer. To use the Biosure^TM^ kit, users draw a blood sample using a capillary lancet, collect the sample in a test stick, push the test stick into a buffer pot, then wait 15 min before interpreting the result using included instructions. For the SELPHI protocol, see Gabriel *et al*. 2018 19.

**Figure 1:**
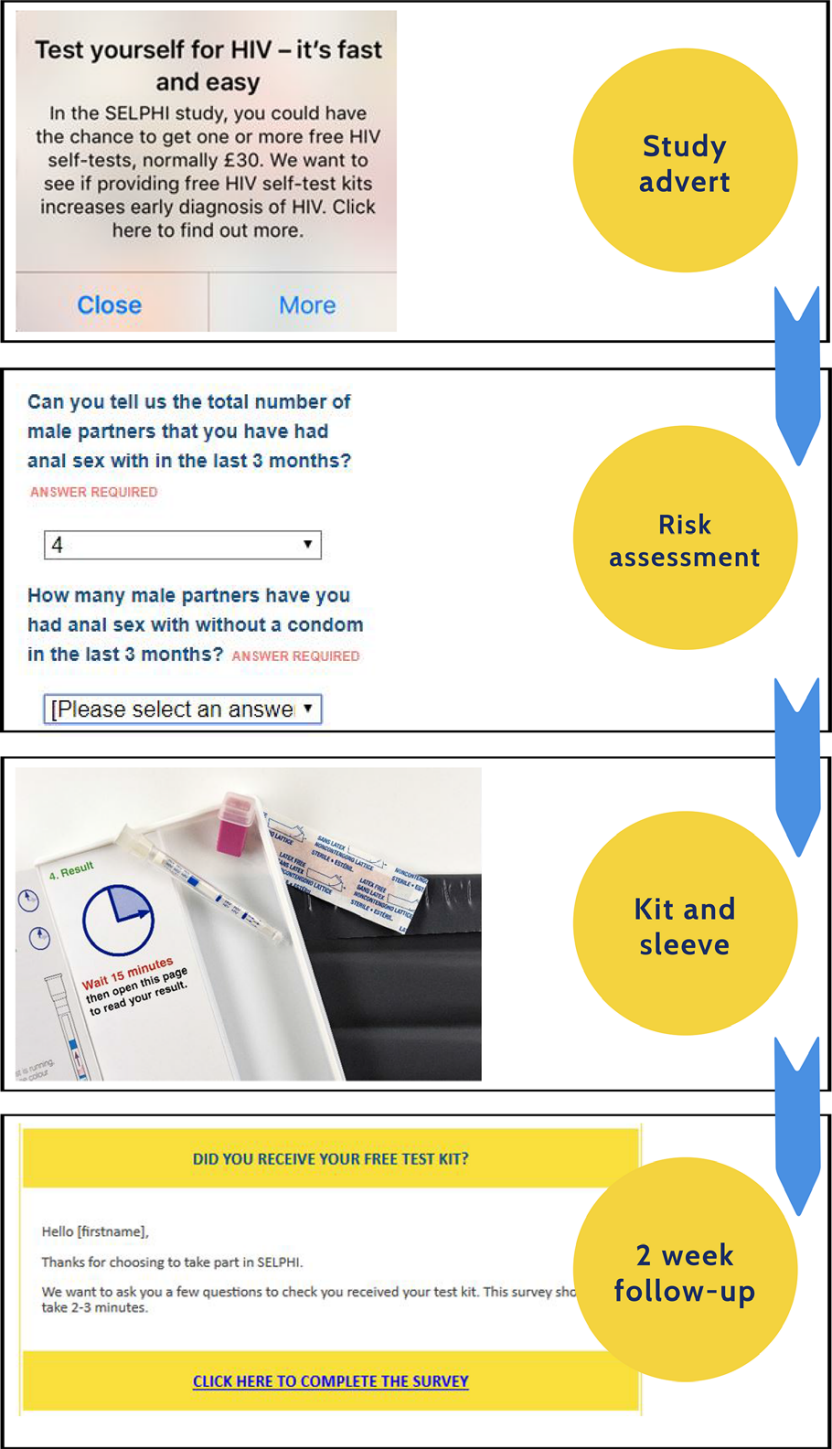
The SELPHI (Self‐Testing Public Health Intervention) intervention A.

**Figure 2:**
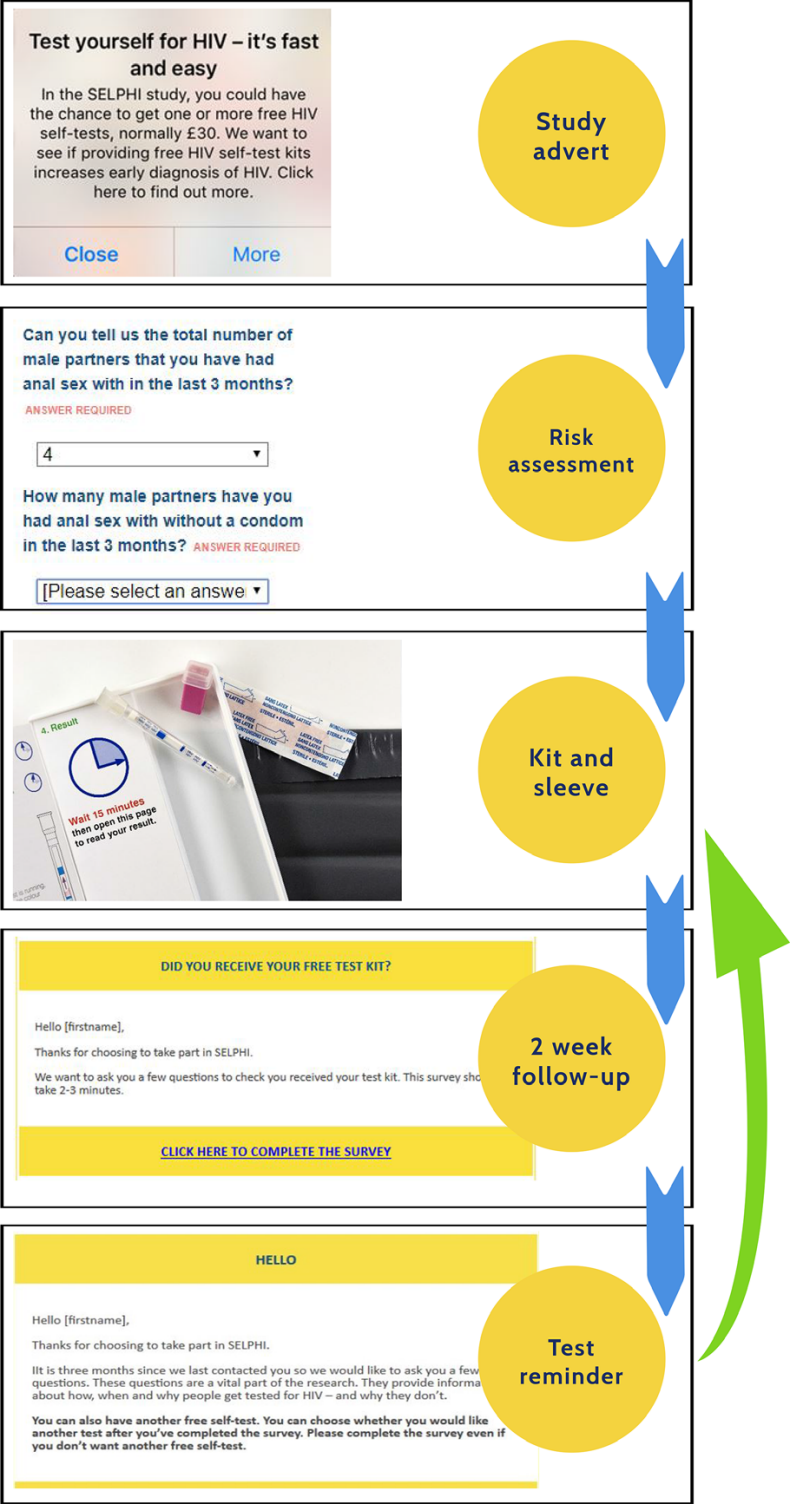
The SELPHI (Self‐Testing Public Health Intervention) intervention B.

The interventions included multiple components which worked together to support uptake of HIVST, continued engagement with testing more broadly and linkage to care if positive (see Fig. 1). Intervention A began with targeted recruitment through adverts on apps and social media designed to increase motivation to test and reduce perceived capability barriers; then a baseline risk assessment (enrolment survey), which collected demographic and behavioural data prior to randomization A in which participants were allocated to being offered an HIVST kit or not. The HIVST kit was then delivered by post directly by the manufacturer. Following kit delivery a 2‐week follow‐up survey was sent via email which asked for confirmation of receipt and use of the kit, the result and provided linkage to care information for those with positive results. All those randomized to receive HIVST in intervention A were entered into randomization B (provided they met eligibility criteria outlined above) and were randomized to SoC or to intervention B, in which HIVST kits were offered 3‐monthly for up to 2 years. They received a test reminder with an embedded risk assessment which was delivered every 3 months, with provision of a further test if desired with an additional follow‐up survey 2 weeks afterwards (see Fig. 2). Both interventions were modelled on what would probably be provided in routine online provision of free kits 14, 15, 20.

This SELPHI substudy addresses critical questions surrounding intervention acceptability and kit usability. The substudy aimed to explore the experience of utilizing HIV self‐tests and the implications for further intervention development and scale‐up. The specific objectives were to understand motivations for accessing HIVST; to explore intervention acceptability; and to characterize experiences of kit use.

## Methods

This qualitative substudy involved 37 semi‐structured interviews with cisgender MSM within the SELPHI RCT. The first 10 interviews took place in May 2017 during the pilot phase, with the remaining 27 conducted during the main trial between January and October 2018.

Interviews were conducted remotely (*n* = 17) or face to face (*n* = 20) depending on location, with a geographical spread of participants across England and Wales. We recruited 25 participants who received intervention A only, 10 who received intervention B and two who reported a positive result (both from intervention A). Purposive sampling ensured diversity in HIV testing experience, age, highest educational qualification (HEQ) and ethnicity. Potential participants who consented to in‐depth interviews were approached by the lead author who provided details about the study and scheduled interviews. The face‐to‐face interviews were conducted in Cardiff and Newcastle to increase the diversity of experience of levels of gay community and social opportunity, as well as smaller scale HIV/STI service infrastructure. Participants provided written or verbal recorded consent and were compensated £30 for their involvement. Only cisgender MSM were included, as a study exclusively with transgender participants is ongoing 21, 22.

The topic guide covered testing history, engagement with SELPHI, experience of the interventions and preferences for future HIVST interventions. It was piloted with two participants, refined, and used for a further eight interviews. Following this, additional questions were added to explore intervention acceptability in greater depth, including a demonstration of the kit and a revisiting of the supportive components of the interventions.

Ethical approval was granted by University College London (UCL) (ref: 11945) and the London School of Hygiene and Tropical Medicine (LSHTM) (ref: 9233/001).

Interviews, all conducted by the lead author, were audio‐recorded and transcribed verbatim. Data analysis followed a Framework approach 23, 24. Our framework drew from theorized key components of intervention acceptability from formative work, the wider literature and systematic reviews 10, 17, 25. This framework was piloted, refined and applied to all transcripts by the lead author.

## Results

The sample of 37 men was diverse with regard to age, education, previous HIV testing history, and number of CAI partners (Table 1). Thirty‐five reported receiving a negative self‐test result and two had received a positive result from their self‐test. Here we outline two broad areas: experience of the HIVST intervention (focussed on acceptability), and experience of using the kit (usability).

**Table 1:** Participant demographic characteristics

Demographic characteristic	Number of participants
Age	
16–25 years	11
26–40 years	16
≥ 41 years	10
Ethnicity	
White	29
Black	3
Asian	2
Other/ mixed	3
Sexual orientation	
Gay	24
Bisexual	5
Don’t use a term	1
Undisclosed	7
Recency of HIV testing	
Never tested	7
≥ 12 months	17
< 12 months	13
Highest educational qualification (HEQ)	
Low*	7
Medium†	11
High‡	19
Number of CAI partners in preceding 3 months	
0	12
1	14
2–3	7
4–10	4
> 10	0
HIVST outcome	
Positive	2
Negative	35
Intervention	
A	27
B	10

CAI, condomless anal intercourse; HIVST, HIV self‐testing.

* GCSEs and below.

^†^ A‐levels or equivalent; higher education below degree level.

^‡^ Degree or higher.

### Intervention experiences

This section relates to participant experiences of the overall intervention. First, we consider the appeal of the interventions, including initial motivations to access HIVST. Secondly, we discuss the acceptability of the psychosocial components embedded in the surveys, and then we discuss support structures, and finally outcomes.

#### Appeal, attraction and engagement

Study adverts were felt to be relevant, engaging and straightforward. These were praised for using simple language and for highlighting that the intervention was free, that the kit was simple to use and that the testing process was quick. For others, the adverts simply highlighted an attractive, more convenient testing opportunity. Those who did not report significant appeal based on the advert usually reported that the advert served as a prompt when they were considering testing anyway.

For those testing in response to a sexual risk event, HIVST provided a new way to access testing, overcoming personal barriers related to stigma and privacy concerns. This was most pronounced for those who had not previously tested, or those disengaged from testing services.

Yes, it was something new, it was giving me the ability to do it in private so I didn’t have to go somewhere I might bump into somebody who knows me. You know clinics, there’s a stigma there. And yes I think it was just the ease of it, the fact that I could do it at home in private on my own but I would get an answer, a yes or a no. (39 year‐old gay man, not previously tested)

Neither of those who received a positive result had previously tested for HIV. They cited barriers related to stigma, geography and inconvenient clinic hours, but felt these issues were resolved by the opportunity to self‐test.

For men testing for reassurance or as part of routine practice, HIVST offered an increase in convenience and a reduction in opportunity cost, overcoming barriers related to inconvenient clinical opening times, poor service quality and distance to services.

An important additional motivation to seek HIVST was curiosity about a new technology. This was often a primary motivator, particularly common among those who had recently tested via another method, but was also reported as a secondary motivator for MSM who had not previously tested for HIV.

The appeal of HIVST was mediated by emotional responses. Self‐testing was perceived as generating either more or less anxiety, relative to other testing methods, primarily based on testing history and motivation for testing. For some infrequent testers with concerns about health care professionals, the personal control provided by HIVST decreased anxiety by placing them at the centre of their decision‐making.

It [getting the result] felt the same as the other times with my doctor… my doctor telling me, except a lot more comfortable […] because I then was the one… I'm now in control. And I suppose the same would've been if it had have been a positive result. I would have been the one in control of going to my doctor and saying, ‘I've gone ahead and done this. It's come back like this and I need you to investigate’. (49 year‐old man, undisclosed sexual orientation, tested in preceding 5 years)

Those with more routine experience of testing tended to describe self‐testing as helpful for reducing anxiety by facilitating testing in a comfortable setting. For some with less HIV testing experience and men who had recent risk, HIVST amplified emotional responses because of the solitary nature of its use.

#### Risk, reflection and recognition

The supportive elements of the interventions (the behavioural questions in the survey, 2‐week follow‐up and testing reminders for those receiving multiple HIVST kits through intervention B) were generally praised for increasing engagement with testing. The follow‐up after receiving a test provided a sense of connection to the trial, and an expectation that supportive action would be taken if a result was positive.

For participants receiving the offer of repeat HIVST, the testing reminders and risk assessments embedded in surveys provided an opportunity for reflection about recent sexual activity. This reflective experience was described either in neutral or positive terms; none felt it was not worthwhile or that it created significant discomfort.

Just made me think a bit harder of the past three months, what I’d been doing. It didn’t make me feel anything like I shouldn’t be asked this […] it just made me think about all the movement I had in the last three months. (27‐year‐old gay man, tested in preceding 6 months)

#### Care, support and follow‐up

This theme relates to the supportive structures within the intervention which facilitated uptake, including accompanying information describing what to do in the event of a positive result and the 2‐week follow‐up survey.

The support structures were largely considered appropriate and in line with expectations. For those with concerns about the dislocation of testing from care, these structures helped to increase intervention acceptability.

I liked the fact that when I opened it, the first thing was the card that fell out and it was, kind of, like, one side was, ‘If you’re negative, great, continue to test, continue to use condoms, continue to have safe sex,’ and then the other side was, ‘If you’re positive don’t worry, we can help you,’ etc. So that was quite comforting. (35‐year‐old gay man, tested more than 12 months ago)

Rather than source support through the intervention structures, most individuals with emotional support needs (regarding both positive and negative results) looked to their social networks, drawing on partners, family and friends. Both men who reported a positive result first spoke to a family member or friend who supported them in seeking confirmatory testing at a clinic.

#### Impacts, outcomes and expectations

Outcomes following testing varied, especially in relation to HIV testing history. Changes in testing behaviour appeared most pronounced among those with less testing experience. For these men, the interventions dramatically increased accessibility, facilitating testing when they would not have tested otherwise. This group also described reductions in barriers to other testing services, partly through an easing of anxiety facilitated by familiarity with testing and partly through increased engagement with services in general.

For many, HIVST increased testing frequency by facilitating testing between clinic visits. This was most pronounced amongst those receiving intervention B, but was described by individuals who received only a single test from intervention A.

Those who did not have well‐established testing patterns felt that they were likely to entirely replace clinic HIV tests with self‐tests as their needs were better met through this means of testing, thus potentially reducing STI testing frequency.

Well, [my behaviour since joining SELPHI] has already changed in a sense that I'm now getting tested every three months, […] even if I don’t receive one through you guys, I can buy it. (27‐year‐old bisexual man, tested in preceding 6 months)

Both men who received a positive result linked to care within 24 h. One had a very low CD4 count indicative of a long‐established infection. Receiving positive results through self‐testing did not diminish intervention acceptability, with both stating self‐testing had ‘saved their life.’

It’s [HIVST] stopped me transmitting it to other people and saved my life. So yes, even though I got my bad news, it’s been a very positive experience. I would recommend anybody do it, anybody who has got concerns, like I had, about going to clinics and stuff like that, get yourself a self‐test. (49‐year‐old gay man, not previously tested, positive result)

Two participants reported negative outcomes related to the interventions. An individual who was receiving repeat self‐tests shared a test with his partner whose family found it, prompting relationship discord. Another participant fainted on using the lancet to draw blood. These experiences did not diminish acceptability; both men stated they would be happy to use self‐tests in the future.

### Test kit usability

This section relates to men’s experiences using the actual kit. First, we explore capability concerns and test errors, then the emotional impact of using the kit. Finally, we describe participant beliefs regarding sensitivity, specificity and accuracy.

#### Capability, cognition and ease of use

For individuals engaging with HIVST, questions around their own capacity to perform the test were commonplace. The majority used the written instructions provided, with a minority accessing online videos.

While most found using the test straightforward, issues with the lancet were common. A further area of concern for many was pressing the test stick into the pot containing the buffer solution. The information provided was felt not to be sufficiently clear, exacerbating confusion about how far to push the test stick in, and concern about breaking it.

This was the confusing part because this is a square and this is round. So I wasn't sure if this was supposed to go there. And looking at this just at first, you are not sure because, okay, this is round, this is a square. And it doesn't fit instinctively there. So this was where I was confused. This was the part that give me a lot of grief. (40‐year‐old gay man, tested in preceding 12 months)

One individual accidentally released the lancet early, but was able to draw blood by using the needle to prick himself. Two individuals were unable to complete their first test, both because of confusion with how to insert the test stick into the buffer pot. Both sourced a replacement kit from the study team.

Participants universally felt that, with increased experience, these issues would not recur. This was also confirmed by participants receiving repeat HIVST kits who reported that increased use enhanced confidence and competence.

#### Anxiety, relief and emotional engagement

The 15‐min interval between completing testing steps and reading the result was nearly universally described as a period of heightened anxiety. This feature of the test provoked emotional responses beyond that experienced waiting for results through other testing opportunities (e.g. at sexual health clinics) for nearly all participants, even those who felt HIVST reduced anxiety overall.

The level of anxiety experienced varied according to testing history and the self‐assessment of risk: those with more testing experience who were testing out of routine tended to be less anxious. For individuals testing following a risk event and for those without established routines (even if low risk), this wait generated profound feelings of vulnerability.

When I start doing the test, no [I didn’t think it could be positive]. When I’d done the test and then I’m waiting I’m convinced I’ve got everything from Ebola to SARS to HIV. So then it’s not until I actually get the result that I’m confident again. But there’s always just that creeping panic that you could have something, because you don’t know. (25‐year‐old gay man, tested in preceding 12 months)

Unsurprisingly, individuals with positive results described the experience as deeply upsetting, but also described the accompanying support information as being appropriate for their needs. Their emotional responses did not diminish intervention acceptability as both expressed great enthusiasm for HIVST.

#### Sensitivity, specificity and beliefs about accuracy

Participants with more testing experience sometimes had questions about HIVST accuracy. This concern was typically about completing the test as well as questions related to reliability of the technology. These concerns were usually dispelled through the support information or upon receiving a negative test result.

While the vast majority of those with negative results trusted that the test outcome was valid (with the exception of one who had made significant test errors), both men with positive results had significant doubts as to whether the result was correct. This was a primary motivator to seek confirmatory testing.

Honestly I was 50/50 on it [accuracy] because I don’t know, I just thought shit, what if I have [HIV] […] it’s not 100% accurate and then I didn’t know. I wanted to get a proper full‐on test. I know this is a proper test as well, but I just wanted a doctor doing it. (21‐year‐old gay man, not previously tested, positive result)

## Discussion

This study explored key dimensions of HIV self‐test intervention acceptability and kit usability among 37 cisgender MSM drawn from a large HIVST RCT. Self‐testing had different appeal depending on previous testing history and their motivations for accessing testing. In nearly all cases, HIVST reduced barriers to testing, which related to either stigma and privacy issues, or convenience and opportunity cost. Supportive intervention components increased testing engagement more broadly. The intervention support structures were adequate, although most support was drawn from social networks. The kit itself was well regarded, with few significant errors. Concerns regarding kit reliability typically resolved following a negative result, but persisted for those who tested positive. Both participants with positive results linked to care within 24 h.

Those without established testing routines and individuals with recent risk concerns found HIVST to induce anxiety, especially the 15‐min interval between using the test and reading the result. This feature produced profound feelings of vulnerability, beyond what would be experienced while testing through a different method. For others, HIVST reduced anxiety relative to other models by putting them in control of the testing process. This underlines the central role of anxiety in HIV testing; anxiety may produce a key testing barrier for many regardless of their risk and testing history, although perhaps at different stages in the process depending on the testing technology and setting.

These findings underline the importance of intervention design in service delivery and the value of formative work with intended beneficiaries. Each component of both interventions (the advertisements, risk assessments, support components and the kit itself) had a specific relationship with acceptability, in most cases overcoming documented HIVST barriers such as lack of support 10, 13, 17, 25.

This qualitative study demonstrates the potential for HIVST to increase testing frequency for frequent and infrequent testers, in line with existing RCT evidence 26, 27. Infrequent testers may access sexual health clinics less often, however, potentially reducing STI testing in this group, also consistent with existing evidence from the USA but contradicting an Australian study 26, 27. Offering bacterial STI self‐sampling alongside HIVST may ameliorate this. The final RCT results will provide crucial evidence regarding this outcome.

Given that those new to testing frequently accessed the intervention in response to risk, it is especially important that clear information regarding test window periods is provided for those with less testing knowledge. The significant distrust of their results reported by both participants with a positive result underlines the importance of clear, supportive information providing an accessible pathway into clinical care no matter the geographical location.

Finally, minor adverse outcomes (fainting; relationship discord) were reported by two participants. Further research into potentially harmful outcomes is required to develop strategies to ameliorate these. This is particularly important given concerns about the potential for harm arising from HIVST despite the lack of evidence to date 10, 12, 28, 29, 30.

## Strengths and limitations

To our knowledge, this is the first European study that has examined self‐testing intervention acceptability solely among actual HIVST users and will be useful for those working with similar groups in similar health care settings. Nevertheless, some limitations are noted.

The majority of our participants reported negative results. Thus, the data pertaining to those with positive results cannot be considered representative of the experiences of others.

All participants chose to participate in an RCT that delivered an HIVST to an address (residential or otherwise) and that collected substantial amounts of personal data, and all of them consented to being interviewed. This sample, therefore, potentially does not include those with the greatest concerns surrounding disclosure of sensitive information about themselves, a group hypothesized to have a heightened need for HIVST 10, 25.

## Conclusions

This study explored how those using self‐tests experience HIV self‐testing and implications for intervention development and scale‐up. Previous testing experience was key in shaping intervention acceptability and test kit usability. Men were motivated to access the intervention because HIVST reduced specific HIV testing barriers related to convenience, stigma and privacy concerns. The intervention was acceptable, with participants expressing an unexpected degree of enthusiasm for self‐testing, including those with positive results and individuals who experienced adverse events.

## Author contributions

RCT design: MMG, SMc, AJR, DTD, ANP, FCL, AS, MGaf, LM, FMB, TCW and PW. Patient and public involvement: RP. RCT implementation: MMG, SMc, AJR, DTD, YCM, ANP, FCL, AS, MGaf, LM, FMB, TCW, PW, DW and JH. Substudy design: TCW, PW, AJR, FMB, AB, SMc and CB. Data collection: TCW. Instrument design: TCW, PW, AB, FMB and AJR. Analysis: TCW. Drafting: TCW, AB and PW. Conceptual input: TCW, AB, PW, AJR, FMB, SMc, DTD, ANP, FCL, AS, MGaf, MMG, FMB, CB and RP. All authors have approved the final manuscript.

## Ethical approval

Ethical approval for the RCT and qualitative substudy was provided by UCL and LSHTM (refs: 11945 and 9233/001). SELPHI was prospectively registered with the ISRCTN registry on 8 October 2016 (ref: ISRCTN20312003). All participants provided informed consent ahead of their involvement in the RCT and qualitative substudy.
